# Experiment Selection in Meta-Analytic Piecemeal Causal Discovery

**DOI:** 10.1109/access.2021.3093524

**Published:** 2021-07-01

**Authors:** NICHOLAS J. MATIASZ, JUSTIN WOOD, WEI WANG, ALCINO J. SILVA, WILLIAM HSU

**Affiliations:** 1 Departments of Bioengineering, Neurobiology, and Radiological Sciences, University of California at Los Angeles (UCLA), Los Angeles, CA 90024, USA; 2 Departments of Computer Science, Neurobiology, and Bioengineering, University of California at Los Angeles (UCLA), Los Angeles, CA 90095, USA; 3 Department of Computer Science, University of California at Los Angeles (UCLA), Los Angeles, CA 90095, USA; 4 Departments of Neurobiology, Psychiatry and Biobehavioral Sciences, and Psychology, University of California at Los Angeles (UCLA), Los Angeles, CA 90095, USA; 5 Departments of Radiological Sciences, Bioinformatics, and Bioengineering, University of California at Los Angeles (UCLA), Los Angeles, CA 90095, USA

**Keywords:** Causal discovery, cause effect analysis, computer aided analysis, design of experiments, evidence synthesis, graphical models

## Abstract

Scientists try to design experiments that will yield maximal information. For instance, given the available evidence and a limitation on the number of variables that can be observed simultaneously, it may be more informative to intervene on variable *X* and observe the response of variable *Y* than to intervene on *X* and observe *Z*; in other situations, the opposite may be true. Scientists must often make these decisions without primary data. To address this problem, in previous work, we created software for annotating aggregate statistics in the literature and deriving consistent causal explanations, expressed as causal graphs. This meta-analytic pipeline is useful not only for synthesizing evidence but also for planning experiments: one can use it strategically to select experiments that could further eliminate causal graphs from consideration. In this paper, we introduce interpretable policies for selecting experiments in the context of piecemeal causal discovery, a common setting in biological sciences in which each experiment can measure not an entire system but rather a strict subset of its variables. The limits of this piecemeal approach are only beginning to be fully characterized, with crucial theoretical work published recently. With simulations, we show that our experiment-selection policies identify causal structures more efficiently than random experiment selection. Unlike methods that require primary data, our meta-analytic approach offers a flexible alternative for those seeking to incorporate qualitative domain knowledge into their search for causal mechanisms. We also present a method that categorizes hypotheses with respect to their utility for identifying a system’s causal structure. Although this categorization is usually infeasible to perform manually, it is critical for conducting research efficiently.

## I. INTRODUCTION

A major goal in science is to identify causal mechanisms. Scientists try to understand, for instance, how cigarettes cause lung cancer, or how a genetic mutation causes memory loss. As suggested by the refrain “correlation does not equal causation,” a causal model not only predicts correlations in a system but also predicts how that system will respond under interventions. This difference between correlative and causal models is particularly crucial for a physician, who tries to cure a patient’s disease with a surgical or pharmacological intervention.

In the last few decades, causality has been formalized using mathematics, yielding the enormously successful model known as a causal Bayesian network, or causal Bayes net [4], [5]. This model represents causality with a *causal graph*, a network of nodes and directed edges (e.g., *X* → *Y*) that correspond to the system’s variables and causal relations. Using this model of causality, researchers have developed *causal discovery* algorithms, which identify the causal graph that describes and predicts the behavior of a system’s variables [6].

There are a variety of causal discovery algorithms that operate on primary data [7]; however, there has been relatively little work on the problem of building causal models with only textual information from scientific communication. This is an important problem because much of the information that a scientist encounters is free text: research articles, for instance, are often unaccompanied by primary data but contain aggregate statistics that should inform a scientist’s understanding of the system.

To address this problem, we previously built a pipeline for *meta-analytic causal discovery*: First, the scientist annotates statistical results in free-text research articles—for instance, using the *research map* representation [8]. Next, these annotations are input to an algorithm that identifies the causal graphs consistent with the annotated results. The scientist can then inspect the consistent graphs to see which inferences arise out of the synthesis of annotated research articles [9], [10].

In this paper, we demonstrate how this meta-analytic approach can inform not only evidence synthesis but also experiment selection. In much of the causal discovery literature, it is assumed that an experiment allows scientists to observe every variable in the system simultaneously. However, this is often infeasible: instead, scientists perform experiments on subsets of the system’s variables and combine the results from these subsets analytically—a technique known as *piecemeal causal discovery* [1]–[3]. This approach is often required in fields like biology due to technological limitations and living organisms’ immense complexity. Piecemeal causal discovery often fails to identify the one true causal graph for the system under investigation, regardless of the number of experiments that can be performed [1]–[3].

In the context of piecemeal causal discovery, we conceive of experiment selection as encompassing two main decisions: (1) the choice of which phenomena—out of all potential phenomena in a system—will be involved in the experiment, and (2) the choice of which empirical strategy will be used: either a passive observation or an intervention where one or more of the phenomena are manipulated. Here we consider studies that each involve two phenomena, where neither or one of the phenomena is intervened on—a widespread occurrence in molecular and cellular biology [8]. For instance, given the available evidence and a limitation on the number of variables that can be observed simultaneously, it may be more informative to intervene on variable *X* and observe the response of variable *Y* than it would be to intervene on *X* and observe *Z*; in other situations—with different evidence available—the opposite may be true. Consider, for example, the following three causal graphs:

1. *X* → *Y, X* → *Z*
2. *X* → *Y, X* → *Z*
3. *X* → *Y, X* → *Z*

If we obtained information that led us to believe the true causal graph was either graph 1 or graph 2, it would be more informative to intervene on *X* and observe whether *Y* covaried, thus allowing us to determine the relation between *X* and *Y*. (Note that graphs 1 and 2 have the same edge relation between *X* and *Z*.) If instead, we obtained information that led us to consider graph 1 and graph 3, we would then prefer the experiment in which we intervene on *X* and observe whether *Z* covaries, as these two graphs have the same edge relation between *X* and *Y*. There are still other situations where, in the presence of conflicting evidence, it could be most instructive to repeat an experiment. These decisions are often left to the subjective judgement of the scientist [11]. A more objective and systematic approach is achieved by representing empirical results with causal graphs.

Causal discovery algorithms will often return not a single causal graph but a set of graphs, each of which equally satisfies the constraints imposed by the input data. This set of consistent causal graphs is known as a *(Markov) equivalence class* [4]. The size of the equivalence class indicates the number of causal explanations that remain viable, given what is known; it thus indicates our degree of ignorance regarding the system. Therefore, an equivalence class not only synthesizes the causal implications of empirical evidence but also provides a formal, hypothesis-generating device for selecting experiments: it encodes precisely which causal relations are determined, and which relations remain underdetermined. For instance, if a set of empirical results is consistent with more than one causal graph—each with its own configuration of edges—a researcher can assess which hypotheses are worth pursuing by inspecting exactly which causal relations remain viable. A causal graph’s underdetermination can thus help researchers to plan experiments by indicating which experiments are needed to fully determine the causal structure of the system.

We characterize this underdetermination with a causal graph’s *degrees of freedom*, which represent the diversity of edge relations that appear in the graphs of an equivalence class [9], [10]. For example, all graphs in an equivalence class may have the same edge relation between the variables *X* and *Y* (e.g., *X* → *Y*), but there may be a diversity of edge relations between the variables *Y* and *Z* (e.g., *Y* ← *Z* and *Y* → *Z*). In light of these options, potential experiments can be chosen based on how much information they would provide—specifically, how much they could distinguish between remaining relations, thus pruning the existing model space of consistent causal graphs. This analysis must be agnostic to the result of each potential experiment, which of course cannot be known in advance. With simulations, we show that experiment selection based on the equivalence class’s degrees of freedom outperforms random experiment selection, in that fewer experiments are needed to identify causal structures. Within the same computational framework, we also demonstrate how to categorize a given hypothesis according to its utility for revealing new causal information regarding the system under investigation.

This paper thus makes the following contributions:

1. Two experiment-selection algorithms with readily interpretable heuristics tailored to meta-analytic piecemeal causal discovery—a setting that is ubiquitous in the biological sciences (§§ III-A–III-B) [34];
2. Simulations of the experiment-selection algorithms that demonstrate (1) trade-offs between computational efficiency and the efficiency of experimentation for causal discovery, as well as (2) inherent limitations of piecemeal causal discovery involving two-variable experiments (§ III-D) [34];
3. A hypothesis-categorization algorithm that guarantees whether an experiment designed to test a given hypothesis could possibly yield new causal information that would further determine a system’s causal structure, given a knowledge base of existing experimental results (§ III-C) [34];
4. A simulation of the hypothesis-categorization algorithm that demonstrates how the proportion of informative and uninformative hypotheses changes as causal-structure information is obtained through experimentation (§ III-D).

## II. BACKGROUND

As part of their investigations, scientists frequently encode their experimental results into either qualitative or quantitative models. These models typically represent correlations between variables, but correlations show only how variables can be expected to change with respect to each other. Correlations do not show which variables *cause* other variables to change. A causal model explicitly represents which variables cause others to change, allowing it to predict how the system will behave if it undergoes an intervention. This predictive ability is useful, for instance, to a biologist who would like to predict how a cell will respond to a chemical, or to a clinician who would like to predict how a patient will respond to a drug.

### A. CAUSAL GRAPHS

A causal model can encode how its variables interact using a *causal graph*—a directed graph, *G* = (**V, D**), where **V** is the set of variables in the model, and **D** ⊆ **V** × **V** is a set of directed edges among the variables in **V**. Relative to the variables in **V**, a directed edge in the graph, *x_i_* → *x_j_*, conveys that the variable *x_i_* ∈ **V** at the tail has a direct causal effect on the variable *x_j_* ∈ **V** at the head [4], [5]. The (*Markovian*) *parents* of a particular variable *x_j_* consist of every variable that has a direct edge from itself to *x_j_*; these parents can be thought of as the “variables Nature must consult before deciding the value of [*x_j_*]” [5]. The simple graph *long-term potentiation* → *spatial learning* encodes not only that long-term potentiation and spatial learning are correlated but also that long-term potentiation *causes* spatial learning. Although a correlation may not imply causation, causation guarantees specific correlations.

A causal graph’s configuration of edges is known as a *causal structure.* This concept helps distinguish between a causal graph and a causal model: whereas a causal graph shows only the causal structure of a system, a causal model includes—in addition to a specific causal structure—a complete parameterization that characterizes the values that each variable takes in relation to others. For example, a causal Bayesian network’s *structure* is expressed as a causal graph; a causal Bayesian network’s *parameterization* is expressed by its conditional probability tables, which reflect the network’s structure [12]. Here we address the task of learning a system’s causal structure, as expressed by a causal graph.

Causal graphs have an enormous model space; the number of unique causal graphs rises super-exponentially with the number of variables. Even if we assume a lack of feedback and thus model a system with a directed acyclic graph (DAG), the number *D* of possible DAGs that exist for *N* variables still grows super-exponentially and is given by the following recurrence relation [13]:

(1) $$D(N)=\sum_{k=1}^{N}{\left(-1\right)}^{k-1}\left(\begin{array}{c} N\\ k \end{array}\right)2^{k(N-k)}D(N-k)$$

This model space is relatively small for small sets of variables: when considering one, two, and three variables in an experimental system, there are one, three, and 25 possible DAGs, respectively. But when considering seven variables, there are about 1.1 × 10^9^—over *one billion*—possible DAGs.

### B. CAUSAL DISCOVERY

Identifying the true causal graph for a system is the goal of a field known as *causal discovery* [6]. Each causal graph that can be drawn—with its unique structure—can be considered a particular explanation for the system that it models. The goal of causal discovery is to find the one causal graph that correctly models the system—i.e., the correct explanation for the system’s behavior. Knowing the true causal graph allows us to predict how the system will behave, including when we intervene on it.

Causal discovery is possible due to *bridge principles*, which “connect what can be observed to the underlying causal structure that generates the phenomena” [14]. The bridge principles we use here are two assumptions known as the causal Markov condition and the causal faithfulness condition. Together, these conditions allow for a relation between independencies in a probability distribution and edges in a causal graph [4]. This relation thus allows us to infer the features of a system’s causal graph based on statistical relations that we derive from studies. For instance, if two variables in a system are statistically dependent, the causal graph that models the system will have certain features, such as one or more specific paths that correspond to this statistical dependence. The rules that these relations follow are given in the theory of *d-separation* [5].

We express causal-structure constraints in the form *X* ⫫ *Y* | **C** ‖ **J**. In this notation, *X* and *Y* are two variables that are statistically independent. This independence may have been inferred by statistically conditioning; the set **C** indicates the variables on which we conditioned to infer the independence. Similarly, the independence may have been inferred from an experiment in which one or more variables were intervened on; the set **J** indicates the variables that underwent experimental intervention when the relation manifested [15]. Both **C** and **J** can be the empty set ( Ø ). When **J** = Ø, an observational (i.e., non-interventional) study is performed in which the variables are passively observed, without intervention. Dependence statements have the same form as independence statements but instead use the “*not*-independent” symbol (⫫). For example, the dependence relation

long-term potentiation⫫spatial learning|Ø‖long-term potentiation,

conveys that long-term potentiation and spatial learning were observed to be correlated in an experiment that intervened on long-term potentiation; in this case, no variables were statistically conditioned on to infer this independence.

The inference from statistical relations to causal graphs is not trivial: a set of (in)dependence relations may imply not just one graph but an equivalence class—a set of multiple graphs that are all equally consistent with the relations. An example of an equivalence class is these three causal graphs:

- *X* → *Y* → *Z*,
- *X* → *Y* → *Z*,
- *X* → *Y* → *Z*.

Each of these causal graphs is equally consistent with the following statistical relations:

- X⫫Y,
- Y⫫Z,
- Z⫫X,
- X⫫Z|Y.

Given a set of (in)dependence relations over a set of variables, it is not immediately obvious which causal graphs are consistent with the relations. In principle, a researcher could derive the equivalence class by hand; however, this manual computation is infeasible for all but the simplest of cases. And causal inference is further complicated by conflicting information. For instance, one experiment may suggest that two variables are dependent, while another experiment may suggest that they are independent. A principled approach to causal discovery should include a method to resolve such conflicts.

### C. CONSTRAINT-BASED CAUSAL DISCOVERY

Here we use an approach known as *constraint-based causal discovery.* The strategy is to express information about a system in the form of logical propositions, which serve as constraints on the causal structure. These constraints then guide how an algorithm searches for the set of causal graphs that are optimal, according to some optimization criterion. Although research articles are often unaccompanied by the primary data that underlie them, articles often contain constraints implicitly in the form of statistical relations, statements indicating either a dependence or independence between two variables. Beyond stating that variables are (in)dependent, an (in)dependence relation may be qualified by additional context: the relation may have been observed only when one or more other variables were statistically conditioned on, or when one or more variables were intervened on—or both.

The particular constraint-based algorithm that we use—developed by [15]—was chosen because it is currently the state of the art in causal discovery. Among current methods, it considers the most general model space: neither acyclicity nor causal sufficiency needs to be assumed—the algorithm can thus consider models that contain both cycles (feedback) and latent confounders. Additionally, the algorithm’s constraint-based approach enables the formalization of background assumptions [6], as well as the degree-of-freedom approach described in the Methods section below.

A scientist who performs experiments to identify the true causal graph is “searching for a needle in a really huge haystack of falsehoods” [16]. An experiment’s result can show the scientists which parts of the haystack are safe to remove: namely, all causal graphs that are inconsistent with the result.^1^ When a result is expressed as an (in)dependence relation, the rules of d-separation can be used to identify the particular causal graphs that are consistent with the result. Any scientist who understands d-separation can use a pen and paper to check whether an (in)dependence relation is consistent with a causal graph. But this computation is infeasible to do manually when there are thousands of possible graphs, as is true even for a system with only five variables. Therefore, the strategy taken by [15] is to have this done computationally.

The algorithm uses answer set programming (ASP), a type of logic programming that is useful for solving very challenging problems such as NP-hard optimization tasks. It is based on the concept of declarative constraint satisfaction [17], [18]. In this context, the constraints are (in)dependence relations, and they are satisfied only by the particular causal graphs that encode those relations, as given by the rules for d-separation.

The algorithm proceeds in the following steps. First, (in)dependence relations among the system’s variables are obtained—either by performing statistical independence tests on the data [15], or by annotating statistical relations that are reported in the literature, as is done with the *ResearchMaps* web application [8]–[10]. If none of the constraints conflict with each other, then a Boolean satisfiability (SAT) solver [19] is sufficient to find the consistent causal graphs [20]. However, if the constraints contain conflicts—for instance, if one constraint states that *X* and *Y* are independent, while another states that they are dependent—then a Boolean *maximum* satisfiability (MaxSAT) solver is required. In this case, each constraint is assigned a weight that denotes its confidence, and the solver finds the causal graphs that minimize the sum of the weights for unsatisfied constraints [19]. Weights can be assigned based on the *p*-values of independence tests [15] or based on other measures of confidence, such as the evidence score for the research map edge from which the constraint was derived (see below). Reference [15] formulated the search for (maximally) consistent causal graphs as a constrained optimization problem: For the (in)dependence constraints **K** over the variables **V**, each with a non-negative weight *w*(*k*), we search through a class of graphs, G, to find the causal graph G* such that

(2) G*∈argminG∈g∑k∈K:G⊨kw(k),

where G⊨k states that the causal structure of *G* does *not* imply the constraint *k*. We thus wish to find the causal graphs that minimize the summed weight of unsatisfied constraints. A state-of-the-art MaxSAT solver named Clingo [21] is guaranteed to converge to a globally optimal solution, thus identifying the one or more causal graphs that maximally satisfy the constraints.

To accommodate both conflicting and conflict-free sets of evidence, here we use the phrase “equivalence class” in two ways: to refer to a Markov equivalence class, as traditionally defined [4]; and to refer to the set of causal graphs that satisfy (2). This second meaning addresses the fact that conflicts can be resolved in multiple ways. Depending on how the conflict is resolved—and which evidence is discarded to achieve this resolution—different sets of graphs will be considered consistent. In this case, the “equivalence class” denotes the set of causal graphs that remain consistent with the evidence that one is currently willing to consider. Unless otherwise specified, we intend this second meaning throughout this text.

### D. DEGREES OF FREEDOM

An equivalence class of causal graphs represents the range of causal interpretations one can defensibly take in light of the available evidence. The diversity of causal structures in an equivalence class represents the extent to which the available evidence is lacking and the extent to which the true causal graph is *underdetermined*: the less evidence there is, the more causal graphs will remain that are consistent with what is known. Because this lack of knowledge is what drives scientific inquiry, quantifying a causal graph’s underdetermination can help scientists to determine which next experiments could be most instructive. We can quantify this underdetermination by considering the diversity of causal structures that exist throughout all graphs in an equivalence class.

The *degrees of freedom* for a causal graph are the possible variations in edge relations that can exist between any two variables throughout an equivalence class [10]. For DAGs, these edge relations are:

- a “left-to-right” edge (*X* → *Y*);
- a “right-to-left” edge (*X* ← *Y*); and
- neither edge (*X* … *Y*).

When we allow for cycles, there is a fourth relation consisting of both directed edges (*X* ⇄ *Y*). Here we consider only three edge relations for DAGs. To fully specify a causal graph over *N* variables, we need to instantiate exactly one of these edge relations for each of the $\left(\begin{array}{c} N\\ 2 \end{array}\right)$ pairs of variables in the graph. Once a particular edge relation is instantiated for a pair of variables (e.g., *X* → *Y*), there are two other possible edge relations—two degrees of freedom—that the pair can take (e.g., *X* ← *Y* and *X* … *Y*). The trivial equivalence class that contains every possible causal graph (satisfying zero constraints) thus has $2\left(\begin{array}{c} N\\ 2 \end{array}\right)$ degrees of freedom. Note that this number is much smaller than the number of possible causal graphs over the same number of variables.

Each causal graph in an equivalence class instantiates these edge relations differently for at least one of the pairs of variables. For each pair of variables in a system, we can determine the number of instantiations that remain underdetermined by looking at the set of all edge relations that appear in a particular equivalence class. In the example of an equivalence class discussed above, the graphs all agree that there is no edge for the pair {*X, Z*}. This edge relation is thus fixed: regardless of which graph is correct, we know that the edge relation for this pair is *X* … *Z*. The graphs in this equivalence class unanimously agree regarding the *existence* of edges for the pairs {*X, Y*} and {*Y, Z*}; however, they do not unanimously agree regarding the edges’ *orientations*. This equivalence class thus has two degrees of freedom. This metric can be expressed as a percentage to convey the amount of underdetermination relative to the number of variables in the system. Returning to the example equivalence class above, there are $2/(2\left(\begin{array}{c} 3\\ 2 \end{array}\right))\approx 33\%$ of the degrees of freedom remaining. Once enough constraints have been supplied to prune an equivalence class to only one graph, zero degrees of freedom remain. This pruning of the equivalence class thus provides an analytic expression of Popper’s conception of science based on falsifiability [22].

### E. RESEARCH MAPS

Experiments in the literature can be represented in various ways; here we use *research maps*, which are graphical representations of empirical evidence [8], [23]–[25]. A research map has two types of information: ontological and methodological.

The ontological information in a research map entails what the experiments showed—e.g., after a gene’s activity increased, a behavior increased. This information is depicted graphically by nodes and directed edges: each node represents a phenomenon involved in a study; each edge represents whether a change was observed between two phenomena. An edge can represent excitation, inhibition, or independence. Excitation and inhibition entail the sign of a correlation—either positive or negative, respectively. Here we simply treat both types of correlation as cases of statistical dependence.

The methodological information in a research map shows the method used to obtain each result—e.g., an intervention in which the quantity of some biological agent was increased, or an observation in which an agent decreased. Symbols on each edge denote the kinds of studies that were performed: the symbols ↑ and ↓ indicate interventions; the symbols Ø^↑^ and Ø^↓^ indicate observations. In each case, the direction of the arrows denotes the direction of the agent’s change. Also, each edge is labeled with an aggregate score that quantifies the recorded evidence. This score, which serves as a cumulative evidence index, is calculated using a Bayesian approach based on evidential convergence and consistency [8].

## III. METHODS

Given a set of (in)dependence relations expressed as constraints on causal structure, we use the causal discovery algorithm discussed above to obtain the degrees of freedom for the equivalence class of causal graphs that are consistent with the constraints. For the case where we assume that the true causal graph is a DAG, the approach is given by Algorithm 1 and proceeds as follows. We define the set **K** as the set of causal-structure constraints obtained for a system with the set of variables **V**. For each {*X, Y*} ∈ **V**, we query the SAT solver once for every degree of freedom that can exist between *X* and *Y*. For a given query, we input the constraints in **K** as well as one additional set of constraints, which encodes the particular degree of freedom being tested. The degrees of freedom *X* → *Y, X* ← *Y*, and *X* … *Y* are encoded by the sets of ASP constraints {edge (X,Y).}, {edge (Y,X). }, and {−edge (X,Y). −edge (Y,X). }, respectively. The hyphens ( – ) in the last set indicate negation to signify that neither edge is present between the nodes. In each run, the SAT solver returns either SATISFIABLE or UNSATISIABLE, indicating whether the degree of freedom appears in at least one causal graph that is consistent with the constraints in **K**. A system with *N* variables and three possible relations between each pair of variables will require $3\left(\begin{array}{c} N\\ 2 \end{array}\right)$ runs of the SAT solver to fully determine the degrees of freedom. Therefore, this procedure splits the set of all possible edge relations into two sets: (1) the degrees of freedom, each of which appears in at least one graph in the equivalence class, and (2) the relations that have been completely ruled out by the constraints. This procedure can be extended to consider cyclic causal graphs by including the degree of freedom indicated by the constraint set {edge (X,Y). edge (Y,X).}.

**FIGURE 1.**
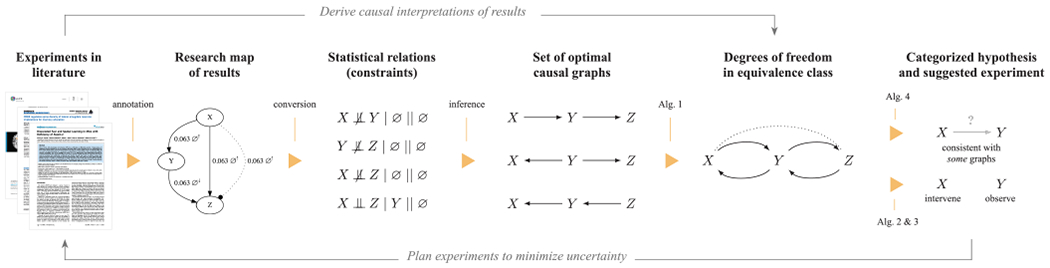
This block diagram provides an overview of the proposed method. Experimental results in the literature are annotated using the research map schema; these results are converted into statistical relations in the form of ASP-encoded causal-structure constraints. An ASP-based causal discovery algorithm then computes the set of causal graphs that maximally accommodate the evidence. Algorithm 1 computes the degrees of freedom for the resulting equivalence class. Algorithm 2 and algorithm 3 are used to identify informative experiments to perform next. Algorithm 4 categorizes hypotheses with respect to their utility for identifying a system’s causal structure.

The degrees of freedom are used as the basis for our experiment-selection methods. We present two methods: the first is based on the degrees of freedom of the equivalence class; the second is based not only on the degrees of freedom but also an expectation metric. The first method is computationally less expensive because it does not require the enumeration of every causal graph in the equivalence class. The second method requires more computation, but its suggestions are correspondingly more informed, leading to more efficient causal discovery. Fig. 1, adapted from [10], provides an overview of the proposed methods. Because of the constraint-based causal discovery algorithm that we use, our approach can readily accommodate the background knowledge from a domain expert [6]. For instance, aside from the constraints obtained from statistical results reported in the literature, a domain expert may be able to articulate other causal-structure constraints that disallow direct edges between certain classes of variables, or that require certain paths involving specific subsets of variables. The ASP encoding that we employ can accommodate virtually any structural constraint that can be imposed on the edges of a causal graph.


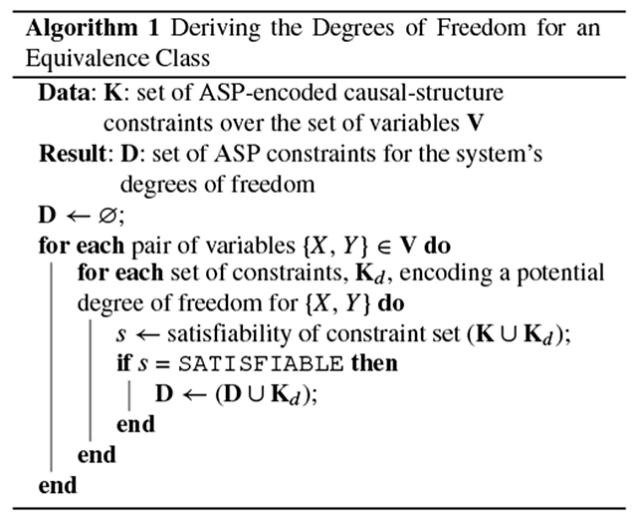


Lastly, we present a method for categorizing hypotheses based on their utility for identifying a system’s causal structure—a process that is usually infeasible to perform manually yet critical for conducting research efficiently.

### A. SELECTING EXPERIMENTS WITH DEGREES OF FREEDOM

Algorithm 2 gives an experiment-selection method based on the degrees of freedom. First, for each pair of variables in the system, {*X, Y*}, we obtain *n*_*X*_,_*Y*_, the number of degrees of freedom in the equivalence class **E** for the pair {*X, Y*}, where *n*_*X*_, _*Y*_ ≤ 2. Next, for the (*X, Y, n*_*X*_, _*Y*_) three-tuple with the largest *n*_*X*_, _*Y*_, we randomly choose one of the suggested experiments for the pair’s degrees of freedom, **D**_*X*_, _*Y*_, as given in Table 1. (If multiple three tuples have the same maximum *n*_*X*_, _*Y*_, we choose one randomly.) The experiments in Table 1 are chosen to be maximally informative, given the degrees of freedom that remain viable. For example, if the relations *X* → *Y* and *X* ⋯ *Y* are the remaining degrees of freedom, we do not suggest an intervention on *Y*, because intervening on *Y* would experimentally control the value of *Y* and thus preclude us from observing a correlation between *X* and *Y* that could arise if an *X* → *Y* relation were present in the true causal graph; intervening on *Y* effectively removes the *X* → *Y* edge, rendering the two degrees of freedom indistinguishable [5]. The suggested experiments are therefore chosen for their ability to distinguish between the remaining degrees of freedom for a given pair of variables. Because this algorithm suggests an experiment given a set of experiments that have already been performed, additional bookkeeping is done to ensure that the experiments are not repeated unnecessarily (see the *while* loop in Algorithm 2). Within the *if* statement, the first condition ensures that if we have multiple competing sets of experiments, we choose the group of experiments that are least well represented in the set **P** (considering all the degrees of freedom, with a preference for the pair(s) of variables with the highest degrees of freedom). The second condition ensures that we choose an experiment from a pair of variables that has at least one experiment that has yet to be run. We enforce an explicit preference for experiments with variables that have not previously been selected. Note that in some edge cases, it is possible for our degrees-of-freedom approach to recommend only experiments that have already been performed. In these rare cases, we randomly choose an experiment that has yet to be run from the pool of all unperformed experiments.

### B. SELECTING EXPERIMENTS WITH DEGREES OF FREEDOM AND EXPECTATION

When it is computationally feasible to compute every causal graph in the equivalence class, we can improve on the efficiency of Algorithm 2: Algorithm 3 gives an experiment-selection method that incorporates an expectation metric. As with Algorithm 2, this method uses the degrees of freedom of the equivalence class. But here the intuition is also grounded in expectation maximization. First, for each pair of variables in the system, {*X, Y*}, and for each possible degree of freedom, *d*, we obtain $m_{X,Y}^{d}$, the number of graphs in the equivalence class **E** that assign the degree of freedom *d* to the pair {*X, Y*}. We use this quantity to calculate the empirical probability of a graph in the equivalence class having that particular degree of freedom: $\frac{m_{X,Y}^{d}}{\left|E\right|}$. We also calculate the number of graphs that would be eliminated from the equivalence class if we learned that this degree of freedom was the actual relation taken by that pair of variables in the true causal graph: $|E|-m_{X,Y}^{d}$. This empirical probability, $\frac{m_{X,Y}^{d}}{\left|E\right|}$, is multiplied by its associated “reward,” $|E|-m_{X,Y}^{d}$, yielding the pair’s expectation for a given $d:e_{X,Y}^{d}=\frac{m_{X,Y}^{d}}{\left|E\right|}(|E|-m_{X,Y}^{d})$. Next, for the $\left(X,Y,d,e_{X,Y}^{d}\right)$ four-tuple with the highest expectation, we randomly choose one of the suggested experiments for *d*, as given in the last three rows of Table 1. (If multiple four-tuples have the same maximum $e_{X,Y}^{d}$, we choose one randomly.) As with Algorithm 2, additional bookkeeping is performed to ensure that experiments are not repeated unnecessarily.


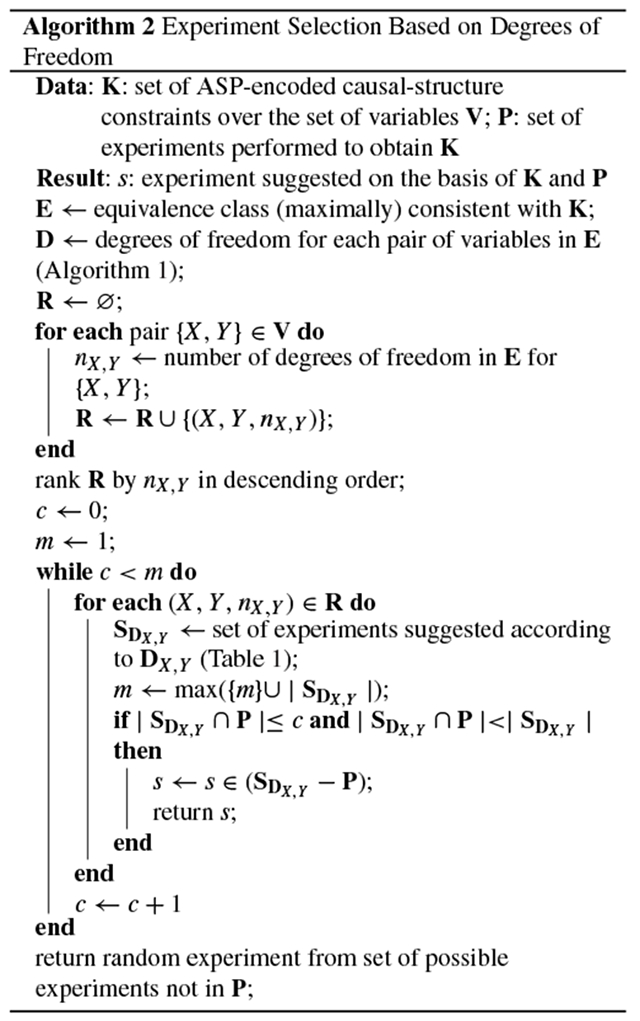


### C. CATEGORIZING HYPOTHESES BY THEIR UTILITY FOR CAUSAL DISCOVERY

Given a knowledge base of constraints on causal structure, we define a method for placing a given hypothesis in one of three categories, with crucial distinctions:

1. *The hypothesis is consistent with **none** of the causal graphs in the equivalence class*. This kind of hypothesis should be pursued only if we are confident that one or more constraints in the current knowledge base are incorrect. The hypothesis is then useful insofar as it identifies which constraints in the knowledge base could be refuted. Otherwise, given the current knowledge base, we would fail to find even one causal graph that is consistent with this kind of hypothesis.
2. *The hypothesis is consistent with **all** the causal graphs in the equivalence class*. Although this kind of hypothesis produces accurate predictions about the system, it is equally unhelpful as the first kind with respect to experiment selection: this hypothesis should not be tested empirically unless we believe there to be a flaw in our current knowledge base and wish to refute one or more of its constraints. The reason is that if a hypothesis is consistent with *all* the causal graphs in the equivalence class, it already follows logically from the knowledge base; the logical proposition that expresses the hypothesis is thus true for all solutions (i.e., causal graphs). In propositional logic, it is said to be in the *backbone* of the satisfying formula [20].
3. *The hypothesis is consistent with **some** (not all) of the causal graphs in the equivalence class*. This kind of hypothesis is most worth pursuing empirically. The experiment’s result—which the current knowledge base cannot predict with certainty—is guaranteed to prune the equivalence class, bringing us closer to the true causal graph.

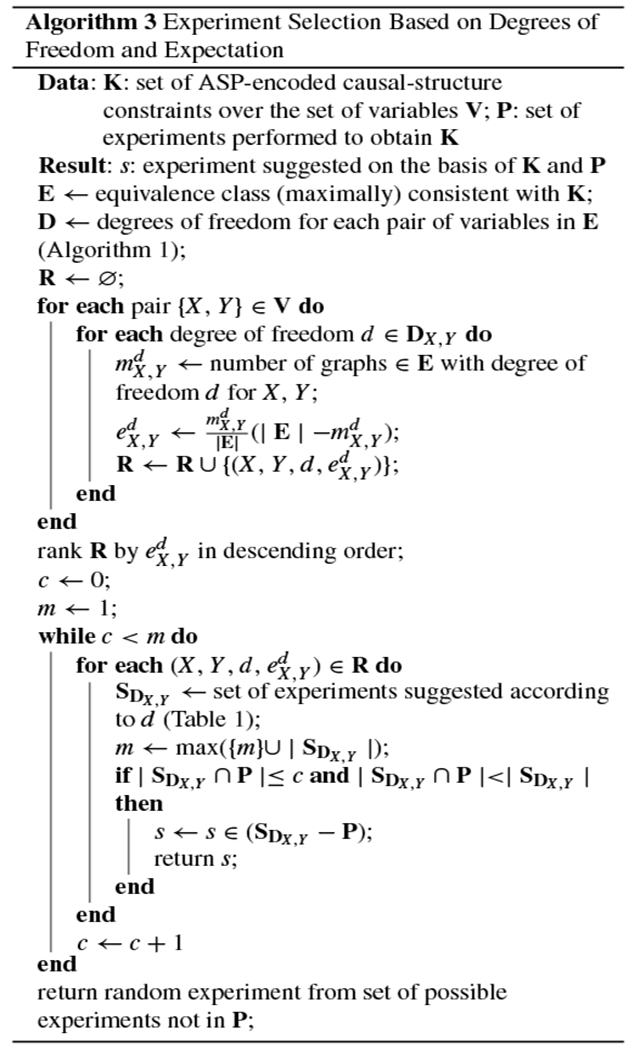


We categorize a hypothesis as follows: First, we express the hypothesis as a formal constraint that can be encoded in ASP; this can be achieved, for example, by adding a hypothetical edge to a research map of empirical results [8]. Second, we query the SAT solver to see whether the hypothetical constraint is consistent with none, all, or some of the causal graphs in the equivalence class. As with the degree-of-freedom analysis, this procedure does not require the SAT solver to perform the expensive computation of enumerating every graph in the equivalence class. Instead, we can simply ask whether the hypothesized constraint is satisfiable, as a binary condition. If the answer is no, then we know that the hypothesis falls into the first category: it is consistent with none of the causal graphs in the equivalence class. If the answer is yes, then we must distinguish between whether the hypothesis is consistent with some or all of the graphs. We do this by querying for the satisfiability of the hypothesis’s negation. If the hypothesis’s negation *cannot* be satisfied by any of the graphs, then we know that the hypothesis falls into the second category: it is consistent with all causal graphs in the equivalence class. If the negation *can* be satisfied by at least one graph, then we know that the hypothesis falls into the third category: it is consistent with some (not all) of the causal graphs in the equivalence class. Therefore, any hypothesis, expressed as a causal-structure constraint, can be categorized with only one or two queries to the SAT solver (Algorithm 4). This categorization of hypotheses can guide experiment selection. Despite the enormous consequences that this categorization has on experiment planning, it is usually infeasible for a scientist to manually compute which category a hypothesis belongs to.


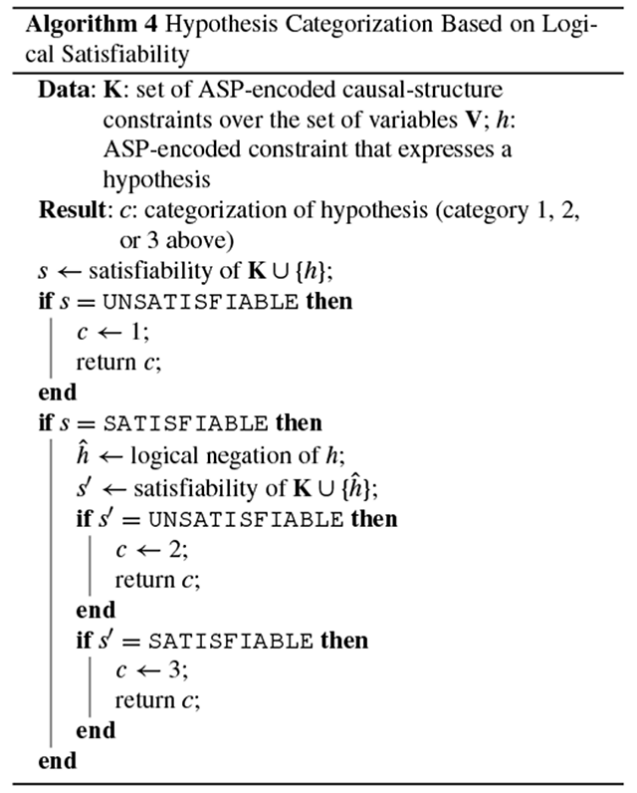


### D. EVALUATIONS

**FIGURE 2.**
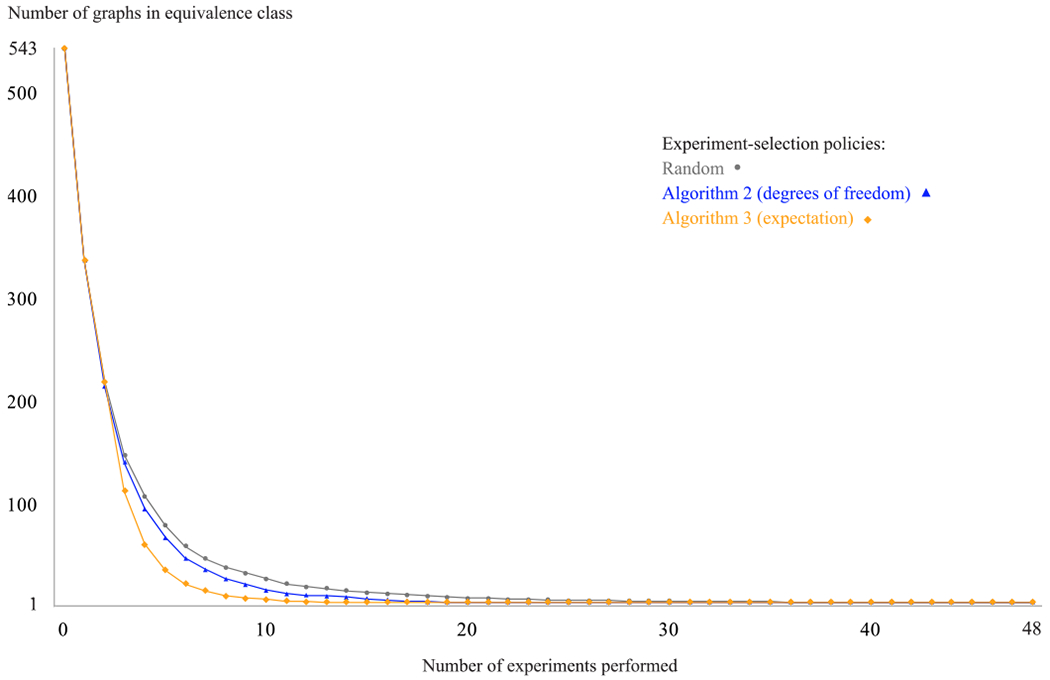
A comparison of three experiment-selection policies: (1) random, (2) Algorithm 2 (degrees of freedom), and (3) Algorithm 3 (expectation). This plot shows the results of the simulation given in algorithm 5 for *N* = 4. The results show the experimental effort that is saved when each experiment is chosen based on the remaining degrees of freedom in the equivalence class.

The experiment-selection policies given in Algorithms 2 and 3 were evaluated using the following simulation, which is given by Algorithm 5. First, one of the 543 possible DAGs over four variables was set as the true graph. Before any experiments were simulated, the equivalence class trivially contained every possible graph. To simulate how researchers learn about a system through repeated experimentation, we sampled study designs according to three different policies: at each iteration, we chose the next experiment (1) randomly, (2) according to Algorithm 2 (degrees of freedom), and (3) according to Algorithm 3 (expectation). The correct result of each experiment was returned by an oracle that assumed causal sufficiency and had access to the true causal graph. Each experiment’s result was added to a growing list of constraints, yielding—at each iteration, and for each experiment-selection policy—an equivalence class of consistent causal graphs. After each experiment, we recorded the number of graphs that remained in each equivalence class. This process continued until we performed every one of the 48 two-variable studies defined by the research map schema. This simulation was repeated for every one of the 543 possible DAGs over four variables, thus showing that the experiment-selection policies are not sensitive to specific features of the true causal graph, such as the density of its edges. For each policy, we then computed the average number of graphs in the equivalence class that remained after each iteration (Fig. 2).


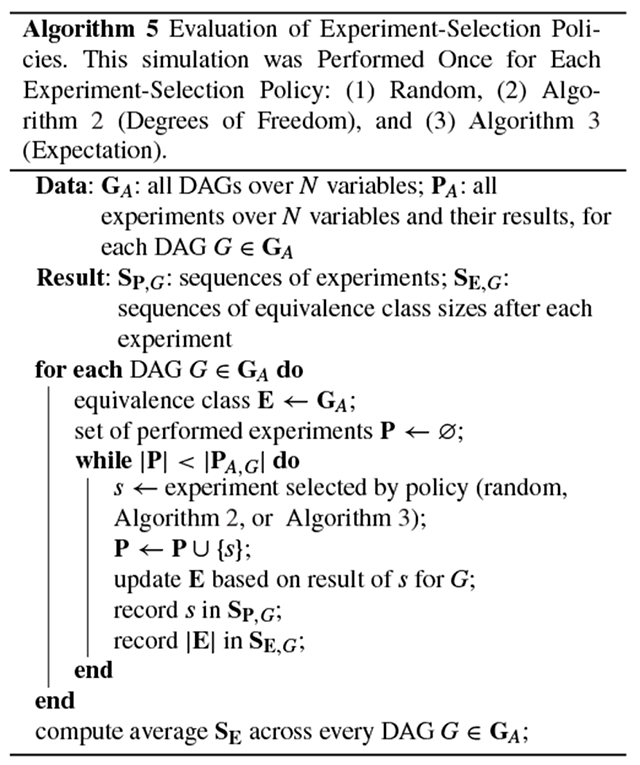


To show how our hypothesis-categorization method can inform experiment planning, we repeated the simulation in Algorithm 5 with an additional step: after each simulated experiment, we categorized the hypotheses implied by the remaining unperformed experiments and recorded the number of hypotheses that fell in each category. For instance, after 10 experiments were performed, 38 two-variable experiments remained to be chosen from, each implying its own hypothesis of independence (or dependence) between two of the variables in the system.^2^ Given the knowledge base of constraints derived from the 10 performed experiments, we categorized each of the untested hypotheses and recorded the number of hypotheses that fell in each category. This process was repeated 543 times—once for each true DAG—and the counts of hypotheses in each category were averaged. The experiments were performed using an Intel Core i5-5250U x64 with 8 GB of RAM.

## IV. RESULTS

The results of the simulations given in Algorithm 5 show that selecting experiments strategically—that is, on the basis of the equivalence class’s degrees of freedom—can save a considerable amount of effort in the laboratory: equivalent levels of underdetermination are reached with far fewer experiments using the suggestions of Algorithms 2 and 3 (Fig. 2). Table 2 shows the number of studies that each experiment-selection policy takes on average to reduce the equivalence class to various sizes. This table highlights that although Algorithm 2 and random selection require only one and two additional studies, respectively, to reach 50 graphs, they require far more studies to reach the minimum average number of graphs achieved by the simulation. Compared to the policy of Algorithm 3, the random policy on average takes 32 additional studies to reach the minimum average value. Algorithm 3 reaches an equivalence class of fewer than 10 graphs—a reasonable number of graphs for a domain expert to review manually—in less than half the number of experiments required by the random policy (9 vs. 19).

**FIGURE 3.**
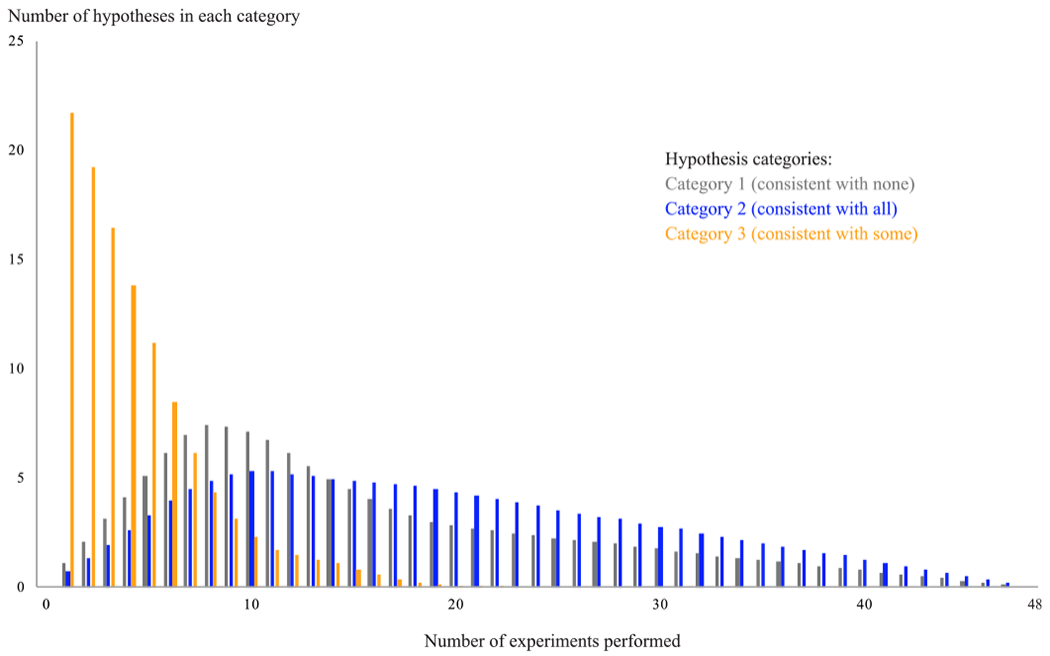
The average number of hypotheses that fell into categories 1, 2, and 3 in a run of the simulation given in Algorithm 5, in which Algorithm 2 was used as the experiment-selection procedure. As each experiment’s result updates the knowledge base of causal-structure constraints, untested hypotheses may change categories, with important implications for the selection of the next experiment.

As expected, Algorithm 3 (expectation) outperforms Algorithm 2 (degrees of freedom), but it does so at the cost of additional computation—a difference that can become quite significant for larger systems [15]. To give a sense of this difference, Table 3 shows the number of ASP models that each experiment-selection policy requires the solver to invoke before suggesting an experiment. Table 4 shows the average runtimes required to complete a single run of the simulations (i.e., for a given true causal graph) presented in Fig. 2 and Fig. 3, respectively, for each of the three experiment-selection procedures. Note that these runtimes reflect the interplay between the speed of each experiment-selection procedure and the additional computation required to consider varying numbers of causal graphs at each simulation step, as the procedures each reduced the equivalence classes at different rates. Comparing the runtimes for Algorithm 2 (degrees of freedom) and Algorithm 3 (expectation) to the runtime of random selection demonstrates that it is worth spending the extra computation time to identify the most informative experiments, in that far less computation is therefore needed to derive subsequent equivalence classes, which are appreciably smaller at each step given the informative experiment that is performed.

Fig. 3 presents the results of our hypothesis-categorization metho’s evaluation, which consist of the averaged counts of hypotheses in each category; Algorithm 2 was used as the experiment-selection procedure in the particular run that is displayed. On average, an appreciable percentage of the hypotheses fall into categories 1 and 2, which are far less informative than category 3 with respect to the goal of identifying a system’s causal structure. As additional empirical results are added to the knowledge base—and the causal structure of the system becomes increasingly determined—the proportion of category 3 hypotheses becomes smaller. In other words, as we learn more about the system, it becomes harder to find informative hypotheses, and easier to make experimental predictions. This is to be expected, as the growing body of empirical results increases our knowledge of the system’s causal structure. A scientist who wishes to determine a system’s causal structure must therefore search for category 3 hypotheses—those represented by the “some” data series in Fig. 3—which is far more feasible using the hypothesis-categorization method presented above. Note that the runtimes for the hypothesis-categorization simulation (Table 4) reflect the time needed to categorize *every* untested, two-variable hypothesis for each of the 48 simulation steps. In real-world applications of the approach, scientists who are deciding whether to pursue a few different hypotheses could obtain their categories in less time.

## V. CONCLUSION

The experiment-selection algorithms that we present are grounded in the type of graphical representation that many scientists—particularly biologists—already use to express causal mechanisms [26]. As a result, scientists can readily interpret the algorithms’ rationale for suggested experiments in the context of the graphical models that they consider to be viable. Although any experiment, if executed properly, can yield useful information regarding a system, strategic experiment selection—even if guided simply by heuristics—can save considerable amounts of work toward identifying a system’s true causal structure. These savings are quantified by the simulations comparing Algorithms 2 and 3 to random experiment selection. Scientists who are constrained to piecemeal causal discovery can thus use these experiment-selection policies to avoid redundant experiments and select instructive ones, examining the degrees of freedom after each experiment to explore the range of edge relations that remain viable. After each empirical result is added to the knowledge base, the suggested experiments should be evaluated with respect to the full diversity of constraints on experiment planning that currently only a human being can consider, including technological limits, research funding, laboratory resources, and investigators’ interests.

The comparison of Algorithms 2 and 3 to random selection does not imply that scientists are currently selecting their experiments at random. Instead, random experiment selection is used to establish a baseline of performance against which other methods can be judged; this approach has precedent in the experiment-selection literature [27]–[30]. Although scientists do not perform their experiments randomly, scientists in most fields do not plan their experiments in perfect coordination. These simulations thus highlight the experimental effort that can be saved when human experiment planning is more globally coordinated and augmented by computational tools—e.g., the ResearchMaps web application [8]—which formalize knowledge in a way that allows for automated inference.

Given that the number of causal graphs grows super-exponentially in the number of system variables, it is impractical to perform for larger systems the exhaustive simulations that we present here. Nonetheless, it is instructive to present the exhaustive simulations performed across all possible true graphs with four variables, as the dynamics of experiment selection can vary tremendously depending on the true graph; the simulations thus show our methods’ average performance across all possible cases.

This approach is particularly helpful given that the limitations and optimal strategies for piecemeal causal discovery have been less well studied compared to the experiment-selection strategies for the general causal discovery setting, in which it is assumed that every variable in the system can be measured in each experiment. In the context of causal discovery, our simulations thus allow for detailed analyses of the limitations of two-variable experiments, which are ubiquitous in the biological literature.

The presented experiment-selection procedures are still beneficial for a variety of real-world research settings. Although scientists regularly study large systems—with hundreds, thousands, or even millions of variables—experiments are often planned to identify relations between small subsets of variables; this is often true, for instance, in molecular and cellular neuroscience: researchers interested in the enormously complex system of the brain will choose to focus on a relatively small number of substructures to understand a particular neural circuit. Our approach can thus be applied iteratively on manageable subsets of variables, allowing researchers to “stitch” together findings to yield new inferences.

For example, in [10] we demonstrate how our degrees-of-freedom method can be used to combine the evidence in two neuroscience articles involving partially overlapping subsets of seven variables. Merging the results of the individual articles yielded a new inference regarding two variables that did not appear together in any of the experiments from the two articles; the resulting inference was deemed plausible by a domain expert.

For large systems, our methods can still render useful results when scientists can afford to wait relatively long amounts of time for supercomputers to return a solution [6]. Given that many experiments in science are very costly, taking months or even years to complete, experiment-selection methods that can save scientists multiple experiments toward identifying a system’s causal structure can still be valuable even if they take days, weeks, or even months to return a result. For yet larger systems that fully exceed the scalability of our experiment-selection methods, researchers could still use our hypothesis-categorization method to evaluate whether a proposed experiment can further determine a system’s causal structure, given a knowledge base of experimental results. Without having to enumerate every graph in the equivalence class, this approach can guarantee whether a proposed experiment will yield information that would reduce the number of viable graphs in the equivalence class.

As we demonstrate in [10], causal-structure information can be latent in the literature, yielding new inferences only when the right combination of findings are merged analytically. Such combinations may be difficult to find, making it impractical for a scientist to know with certainty whether a proposed experiment would yield information that is not already latent in the literature. Thus, if Algorithm 4 categorizes a proposed hypothesis in either the *none* or *all* categories, scientists can know with certainty that their existing evidence is sufficient to specify the outcome of the experiment that would test the proposed hypothesis.

The results of our simulations illustrate a few key points about the limitations of piecemeal causal discovery and the importance of planning experiments in light of the causal explanations that remain viable. It is known that log(*N*) + 1 experiments suffice to identify the true, causally sufficient DAG over *N* variables, where in each experiment, scientists can observe every variable in the system, and intervene on any number of variables in the system. If we are limited to single-intervention experiments, *N* − 1 experiments are sufficient and in the worst-case necessary [31], [32]. Under these assumptions, log_2_(4) + 1 = 4 − 1 = 3 experiments suffice to identify the true DAG over the four variables considered in our simulations. But the experimental context we consider here is further constrained: we consider studies in which only two variables are observed simultaneously and at most one variable can be intervened on per experiment. Thus, on average, between four and five graphs remain in the equivalence class after every possible two-variable experiment has been performed. Our policies’ inability to uniquely identify some of the true causal graphs is in part a manifestation of the limits on piecemeal causal discovery [2], [3], [33]. In future work, it would be instructive to better characterize how the efficiency of causal discovery improves as larger subsets of the system can be observed and intervened on simultaneously. Understanding exactly how much information is lost due to piecemeal causal discovery could help scientists to prioritize the development of laboratory equipment, including technologies that would allow for simultaneous observation of, and intervention on, larger sets of phenomena.

## Figures and Tables

**TABLE 1.** The experiments that would be most informative with respect to a pair of variables, given their particular degree-of-freedom pattern in an equivalence class. These suggested experiments inform the experiment-selection method given in Algorithms 2 and 3. The set J indicates which variables are intervened on in each experiment; when J = Ø, a passive observation of the two variables is performed.

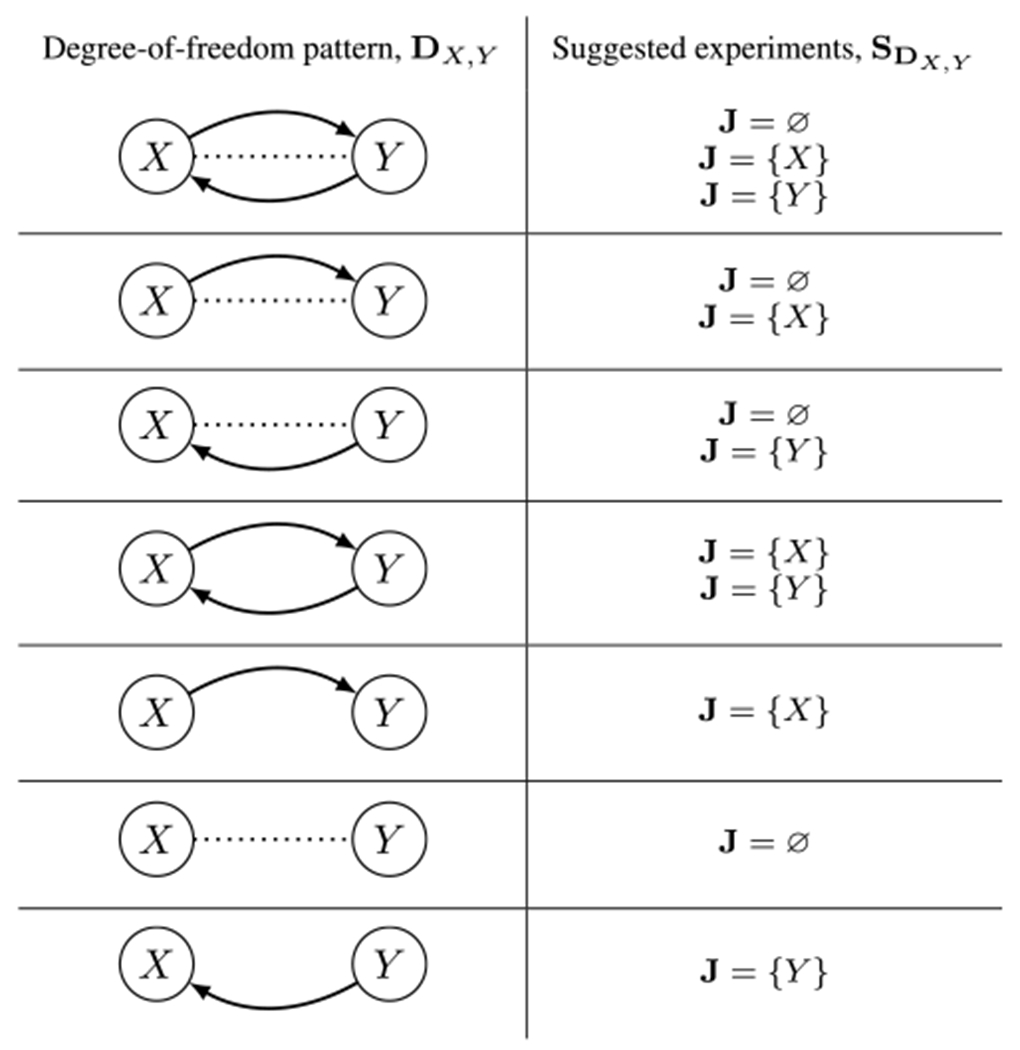

**TABLE 2.** Empirical efficiency of experiment-selection policies.

	Number of studies needed to reach:		
Policy	< 50 graphs	< 10 graphs	minimum
Algorithm 3	5	9	15

Algorithm 2	6	14	23

Random selection	7	19	47

The number of studies that each experiment-selection policy takes on average to reduce the equivalence class to a given size.

**TABLE 3.** Computational efficiency of experiment-selection policies.

	Number of ASP models invoked for:		
Policy	4 variables	8 variables	14 variables
Algorithm 3	543	~ 10^11^	~ 10^36^

Algorithm 2	18	84	273

Random selection	0	0	0

The number of ASP models that each experiment-selection policy requires the solver to invoke in order to suggest an experiment.

**TABLE 4.** Runtimes for experiment-selection and hypothesis-categorization simulations.

	Average execution time (s) to determine:	
Policy	Graphs/equivalence class	Hypotheses/category
Algorithm 3	61.7	246.5

Algorithm 2	34.0	527.7

Random selection	827.3	1001.5

The average runtimes required to complete a single run (i.e., for a given true causal graph) of the simulations presented in Fig. 2 and Fig. 3, respectively, for each of the three experiment selection procedures.

## Footnotes

1 Of course, an erroneous result can mislead the scientists by motivating them to remove a part of the haystack that in fact contains the needle (i.e., the true causal graph). Here we do not model scientists’ fallibility; instead, the focus is on how to reason with evidence and plan experiments, assuming that those experiments will be performed competently.

2 For the simulation, each untested hypothesis assumed an independence relation; had we chosen to assume a dependence for each hypothesis, the counts for categories 1 and 2 would simply be exchanged. The effect of this choice is limited by averaging over all DAGs. What is most noteworthy is the proportion of hypotheses in category 3 to the proportion in either category 1 or 2.
